# Asphericity derived from [^18^F]FDG PET as a new prognostic parameter in cervical cancer patients

**DOI:** 10.1038/s41598-023-35191-8

**Published:** 2023-05-24

**Authors:** Paulina Cegla, Frank Hofheinz, Ewa Burchardt, Rafał Czepczyński, Anna Kubiak, Jörg van den Hoff, Pavel Nikulin, Agnieszka Bos-Liedke, Andrzej Roszak, Witold Cholewinski

**Affiliations:** 1 Department of Nuclear Medicine, Greater Poland Cancer Centre, Garbary 15, 61-866 Poznan, Poland; 2 Helmholtz-Zentrum Dresden-Rossendorf, Institute of Radiopharmaceutical Cancer Research, Dresden, Germany; 3 Department of Electroradiology, Poznan Univeristy of Medical Science, Poznan, Poland; 4 Department of Radiotherapy and Gynaecological Oncology, Greater Poland Cancer Centre, Poznan, Poland; 5 Department of Endocrinology, Metabolism and Internal Disease, Poznan University of Medical Science, Poznan, Poland; 6 Department of Nuclear Medicine, Affidea Poznan, Poland; 7 Greater Poland Cancer Registry, Greater Poland Cancer Centre, Poznan, Poland; 8 Department of Biomedical Physics, Adam Mickiewicz University, Poznan, Poland

**Keywords:** Cancer, Medical research, Oncology, Risk factors

## Abstract

The objective of this study was to assess the prognostic value of asphericity (ASP) and standardized uptake ratio (SUR) in cervical cancer patients. Retrospective analysis was performed on a group of 508 (aged 55 ± 12 years) previously untreated cervical cancer patients. All patients underwent a pretreatment [^18^F]FDG PET/CT study to assess the severity of the disease. The metabolic tumor volume (MTV) of the cervical cancer was delineated with an adaptive threshold method. For the resulting ROIs the maximum standardized uptake value (SUV_max_) was measured. In addition, ASP and SUR were determined as previously described. Univariate Cox regression and Kaplan–Meier analysis with respect to event free survival (EFS), overall survival (OS), freedom from distant metastasis (FFDM) and locoregional control (LRC) was performed. Additionally, a multivariate Cox regression including clinically relevant parameters was performed. In the survival analysis, MTV and ASP were shown to be prognostic factors for all investigated endpoints. Tumor metabolism quantified with the SUV_max_ was not prognostic for any of the endpoints (p > 0.2). The SUR did not reach statistical significance either (p = 0.1, 0.25, 0.066, 0.053, respectively). In the multivariate analysis, the ASP remained a significant factor for EFS and LRC, while MTV was a significant factor for FFDM, indicating their independent prognostic value for the respective endpoints. The alternative parameter ASP has the potential to improve the prognostic value of [^18^F]FDG PET/CT for event-free survival and locoregional control in radically treated cervical cancer patients.

## Introduction

Cervical cancer is a global health problem and ranks fifth place in the incidence and mortality of cancer diseases worldwide^1^. The most common histological types of cervical cancer are squamous cell carcinoma (SCC, 70%) and adenocarcinoma (AC, 25%)^2^. Despite the progress in diagnosis and therapy, this type of cancer still remains a major problem worldwide^1,2^. Beside stage, also age, tumor pathology, size of the primary tumor, lymph node status as well as human papillomavirus (HPV) infections have been shown to be prognostic factors in cervical cancer patients^3^. Imaging modalities used in gynecological malignancies usually are limited to transvaginal ultrasound, computed tomography (CT), and magnetic resonance imaging (MRI).

An increasing role of positron emission tomography/computed tomography (PET/CT) in assessing the severity of the disease or recurrence in gynecological malignancies including cervical cancer has been observed in recent years^4^. Moreover, it has been shown that PET/CT with the commonly used radiotracer ^18^F-fluorodeoxyglucose ([^18^F]FDG) is a useful technique in determining severity of the disease and assessing therapy response in cervical cancer patients^5,6^. The association between [^18^F]FDG PET-derived parameters, like maximum standardized uptake value (SUV_max_), mean standardized uptake value (SUV_mean_), metabolic tumor volume (MTV) and total lesion glycolysis (TLG) and overall survival (OS) or treatment failure are of special interest^7^. However, there are some studies indicating that primary tumor SUV_max_ has no prognostic impact^8,9^. In a recent study, we have investigated the prognostic value of PET in a group of 508 cervical cancer patients^5^. The only PET parameter which showed a significant prognostic effect was the MTV. However, SUV_mean_ and SUV_max_ did not show any prognostic value.

One cause of this insufficient performance could be the well-known shortcomings of SUV quantification, e.g. its dependence on the uptake time, interstudy variability of the arterial input function (AIF), and susceptibility to errors in scanner calibration^10–12^. All of these factors adversely affect the reliability of the SUV as a surrogate of the metabolic rate of glucose consumption. In several publications, it has been demonstrated that the uptake time normalized tumor to blood SUV ratio (standardized uptake ratio, SUR) essentially removes most of these shortcomings and shows an improved correlation with the metabolic uptake rate^13–15^, an improved test–retest stability^16^, and a significantly better prognostic value compared to the tumor SUV^17–20^.

Another proposed PET-derived parameter is asphericity (ASP)^21,22^. ASP reflects the shape irregularity of the primary tumor’s metabolic volume. Our group and others have identified ASP as prognostic factor in several tumor entities^23–26^. In all these studies, ASP proved its ability to improve the prognostic value of PET notably.

The present investigation addresses the question whether the distinctly improved prognostic value of ASP and SUR reported in the above-mentioned publications is also present in patients with cervical cancer. For this purpose, we evaluated ASP and SUR in the same data set as used in our earlier publication^5^.

## Results

Patient and tumor characteristics are shown in Table 1 while summary statistics for the investigated PET parameters are shown in Table 2.

**Table 1:** Patient and tumor characteristics.

Characteristics	Value
Age (years)	
Mean ± SD	55 ± 12
Median	57
Histology	
SCC	455 (89.6)
AC	53 (10.4)
Grading	
n/a	140 (27.6)
G1	28 (5.5)
G2	251 (49.4)
G3	89 (17.5)
FIGO stage	
I	59 (11.6)
II	150 (29.5)
III	250 (49.2)
IV	49 (9.7)
Therapy	
Hyperthermia	264 (52)
Chemotherapy	445 (87.6)
Teleradiotherapy	425 (83.7)
Brachytherapy	396 (78)
Hysterectomy	57 (11.2)
RTCH	402 (79.1)

*SD* standard deviation, *SCC* squamous cell carcinoma, *AC* adenocarcinoma, *n/a* not applicable, *FIGO* International Federation of Gynecology and Obstetrics, *RTCH* radiochemotherapy.

**Table 2:** Summary statistics for the analyzed PET parameters.

Parameter	Mean ± SD	Median	IQR	Range
MTV (ml)	27 ± 28	19.7	9.34–33.9	1.41–223
TLG (ml)	248 ± 321	149	59.6–295	2.96–3120
SUV_max_	12.9 ± 6.17	12.0	8.91–15.6	3.27–50.7
SUR	10.3 ± 4.91	9.61	7.04–12.7	2.61–38.1
ASP (%)	25.8 ± 21.9	18.5	11.6–32.5	0.13–165

*MTV* metabolic tumor volume, *TLG* total lesion glycolysis, *SUVmax* maximum standardized uptake value, *SUR* standardized uptake ratio, *ASP* asphericity, *SD* standard deviation, *IQR* interquartile range.

The majority of patients (n = 402) was treated with radiochemotherapy (RTCH) as primary treatment. In patients subjected to hysterectomy as the primary treatment, an adjuvant treatment (chemotherapy or radiotherapy or radiochemotherapy) based on the risk factors was applied.

Univariate Cox regression using the metric PET parameters revealed ASP as significant prognostic factor for all four endpoints. SUR showed a trend for significance for LRC and FFDM (Table 3).

**Table 3:** Univariate Cox regression.

Parameter	HR	95% CI	P-value	HR	95% CI	P-value
		EFS			OS	
SUR	1.02	0.99–1.06	0.1	1.02	0.99–1.06	0.25
ASP	1.01	1.01–1.02	< 0.001*	1.01	1.01–1.02	< 0.001*
		LRC			FFDM	
SUR	1.05	1–1.1	0.066	1.05	1–1.1	0.053
ASP	1.02	1.01–1.03	< 0.001*	1.01	1–1.02	0.036*

PET parameters were included as metric parameters.

*Statistically significant differences.

*HR* hazard ratio, *CI* coefficient interval, *EFS* event free survival, *OS* overall survival, *LRC* locoregional control, *FFDM* freedom from distant metastases, *ASP* asphericity, *SUR* standardized uptake ratio.

From clinical parameters in univariate Cox regression analysis FIGO stage > II, remained significant for OS (p < 0.001), EFS (p < 0.001) and FFDM (p = 0.029), while hyperthermia and brachytherapy showed significance in EFS (p = 0.001; p < 0.001, respectively), OS (p < 0.001, p < 0.001, respectively) and LRC (p = 0.037, p < 0.001, respectively). All other investigated clinical parameters did not show a significant effect for any of the four endpoints (see Ref.^5^).

After binarization, ASP remained a significant factor for all endpoints. SUR showed a significant effect only for LRC. All results of this analysis are shown in Table 4 which also lists the applied cutoff values are.

**Table 4:** Univariate Cox regression.

Parameter	Risk	HR	95% CI	P value
EFS				
MTV	> 10.4 ml	2.57	1.67–3.97	< 0.001*
ASP	> 35%	2.31	1.68–3.18	< 0.001*
OS				
MTV	> 12.7 ml	2.8	1.75–4.48	< 0.001*
ASP	> 44.4%	2.13	1.4–3.26	< 0.001*
LRC				
MTV	> 13.7 ml	2.82	1.42–5.61	0.003*
SUR	> 12.6	2.13	1.23–3.69	0.0068*
ASP	> 35.3%	3.41	1.99–5.85	< 0.001*
FFDM				
MTV	> 10.4 ml	5.04	1.82–13.99	0.002*
SUR	> 14.3	1.78	0.93–3.4	0.081
ASP	> 34.3%	2.7	1.54–4.72	< 0.001*

PET parameters were included as binarized parameters.

*statistically significant differences.

*HR* hazard ratio, *CI* coefficient interval, *EFS* event free survival, *OS* overall survival, *LRC* locoregional control, *FFDM* freedom from distant metastases, *ASP* asphericity, *SUR* standardized uptake ratio, *MTV* metabolic tumor volume.

For comparison, also the previously published results for MTV (the only significant PET parameter in this investigation) are listed.

**Figure 1:**
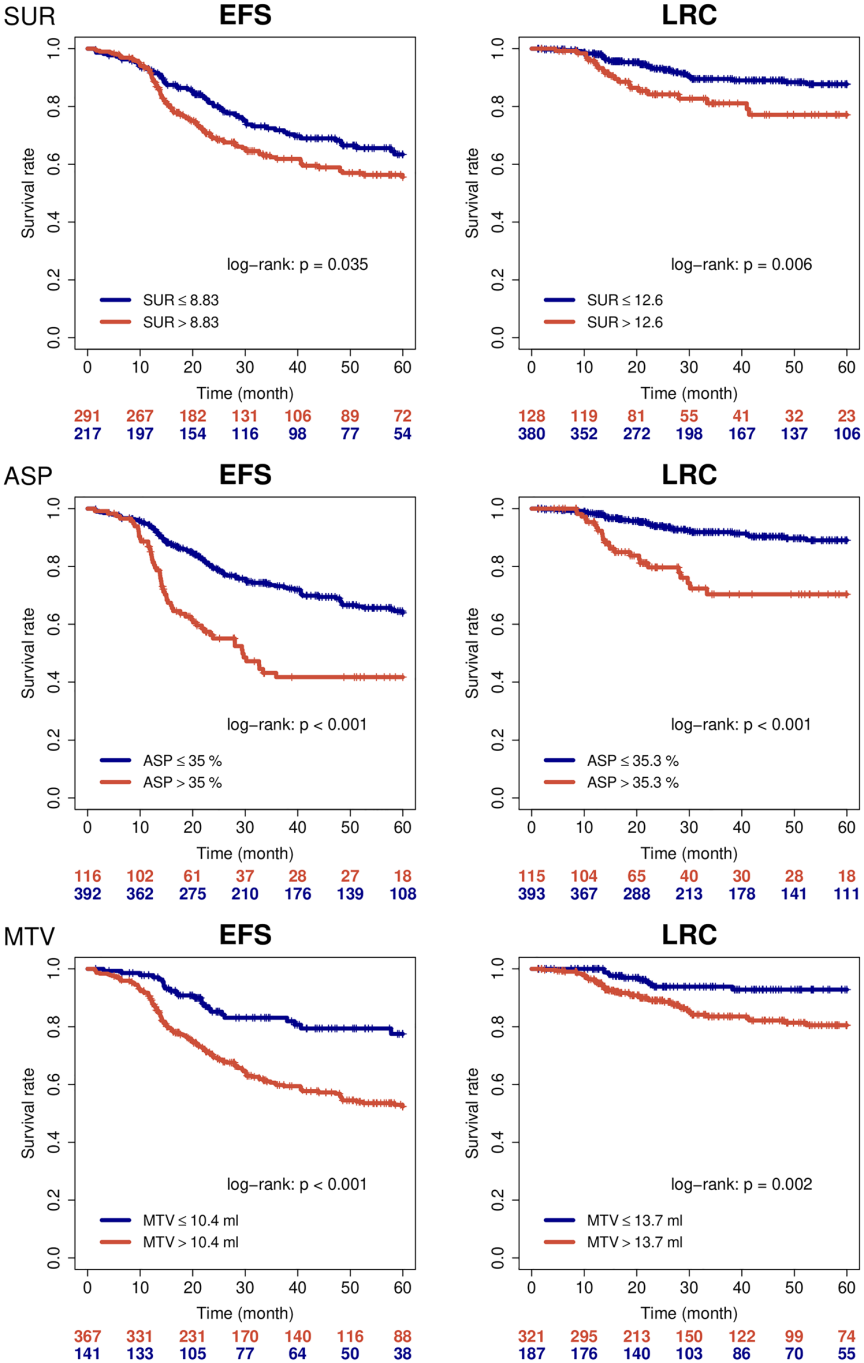
Kaplan–Meier curves for SUR, ASP and MTV with respect to EFS (top) and LRC (below).

In the multivariate analysis with the clinical parameters: hyperthermia, brachytherapy and FIGO stage (see analysis in Ref.^5^) as confounding factors, ASP showed a significant effect for EFS (p = 0.028) and LRC (p = 0.003). SUR was significant for LRC (p = 0.02). However, in a multivariate analysis including both parameters—ASP and SUR—only ASP remained significant (p = 0.01). Corresponding Kaplan–Meier curves are shown in Fig. 1 (the previously published results for MTV^5^ are displayed for comparison).

Stability of the cutoff values was tested for ASP and the two endpoints EFS and LRC. For EFS this test revealed a mean HR of 2.35 (p < 0.001). The cutoff led in all samples to a significant result. For LRC the fraction of significant samples was 99% (mean HR 3.56, p = 0.0036).

## Discussion

In this study, we extended our earlier investigation of patients with radically treated cervical cancer by the evaluation of two further PET parameters, namely ASP and SUR. Our main result is that ASP is an independent prognostic factor for EFS and LRC and notably improves the prognostic value of PET with respect to LRC (HR = 3.41 compared to HR = 2.82 for MTV, the only significant prognostic factor in our previous study). This can be seen as an indication that ASP in general has the potential to increase the prognostic power of PET, which is in agreement with the existing literature. E.g. it was shown that a high value of ASP is associated with worse prognosis and higher stage of the disease^21,22^. In a study of a group of 131 invasive ductal carcinoma (IDC) of the breast, Jung et al. showed that ASP was an independent prognostic factor for progression free survival (PFS) in these patients^27^. A similar investigation was performed by Cheng et al. on a group of 147 oropharyngeal squamous cell carcinoma patients in whom the prognostic value of pretherapeutic [^18^F]FDG PET parameters was correlated with PFS^28^. They found that several PET parameters (including ASP) were significantly associated with PFS.

The prognostic role of ASP was examined also in other aspects. Whi et al. found a relationship of EGFR mutation to SUV_max_, MTV and ASP in lung adenocarcinoma^29^, while Apostolova et al. showed a correlation of Ki67 index and ASP in head and neck cancer patients^21^. Unfortunately, we were unable to examine the correlation between HPV status and Ki67 with ASP, because these laboratory tests were incomplete.

Compared to ASP, our results for SUR are less clear. On the one hand, SUR showed a prognostic effect, independent from clinical parameters, for LRC while SUV does not show any significant effect in this patient group^5^. This shows that the increased correlation of SUR with the metabolic trapping rate compared to SUV, as reported in Refs.^13,14^, directly translates into an increased prognostic value also in this patient group. On the other hand, SUR did not add prognostic information to MTV or ASP. A similar effect was already seen in Ref.^20^ where SUR was investigated in patients with esophageal cancer where the pretherapeutic SUR was a prognostic factor independent from clinical parameters but did not add prognostic information to MTV. Taking into account the rather low effect size of SUR compared to MTV or ASP (Table 4), it seems thus not very likely that pretherapeutic SUR can play a diagnostic role in patients with cervical cancer.

The major limitation of our study is that it is a retrospective single-centre analysis on a group of heterogeneously treated patients. But to the best of our knowledge, it is the first investigation of the prognostic value of the [^18^F]FDG PET-derived parameters ASP and SUR in cervical cancer patients with a substantial follow-up period. Another limitation is that we were unable to investigate the relationship between [^18^F]FDG PET-derived parameters and clinicopathological features, like HPV status and Ki67 index. Finally, we investigated only primary tumor parameters, not including data from metastatic lymph nodes or distant metastases. We intend to overcome all these limitations in a planned comprehensive multicenter analysis.

## Materials and methods

### Patient characteristics

The retrospective, single-center analysis was performed on a group of 508 previously untreated histologically proven cervical cancer patients admitted to the Department of Radiotherapy and Gynecological Oncology between May 2009 and May 2020. Inclusion criteria were: histologically proven cervical carcinoma, chemoradio or radiotherapy with curative intend, and [^18^F]FDG PET prior to therapy. Exclusion criteria were as fellow: recurrence of previous cervical cancer, histology different than SCC or AC, distant metastases (M1 feature). Further details can be found in Ref.^5^ were the same patient group was analyzed. All patients gave their informed consent to PET/CT acquisition as well as for the proposed treatment. Because of the retrospective nature of this study it there was no influence on the treatment decision in the analyzed group. All methods were performed in accordance with the relevant guidelines and regulations. Ethical review and approval (Bioethics Committee at Poznan University of Medical Sciences) as well as patient informed consent were waived for this study, due to the retrospective nature of this study.

### Determination of prognosis

A medical chart review was performed to obtain the follow-up data. Moreover, data obtained from the regional Greater Poland Cancer Registry and the National Cancer Registry was used to estimate the patients’ prognosis. Both institutions are equipped with appropriate data collection procedures to assess the vital status of the patients and possess high quality epidemiological data. The majority of patients was diagnosed with stage III (49.2%) and II (29.5%), respectively, according to the International Federation of Gynecology and Obstetrics (FIGO) classification. Stage I was diagnosed in 11.6%, stage IV in 9.6% of all patients. The most common histological type SCC was diagnosed in 89.6% of patients and AC was diagnosed in the remaining 10.4%. For more details see Ref.^5^.

### [^18^F]FDG PET acquisition

All patients underwent an [^18^F]FDG PET/CT investigation before treatment. Scans were performed 60 ± 15 min after IV injection of [^18^F]FDG with a mean activity of 364 ± 75 MBq. The acquisition was performed using a 16-slice multidetector scanner (Gemini TF 16 Scanner, Philips, Healthcare Medical Systems, Inc, Cleveland, OH) using the following parameters: 100–250 mAs, 120 kV, slice thickness 5 mm. The PET scan was performed in 3D mode with an acquisition time of 1.30 min per bed position (eight-twelve bed positions) covering the same field as the CT scan. The obtained images were reconstructed using the ordered subset expectation maximization (OSEM) iterative algorithm. All studies were performed on the same scanner to provide consistent reconstruction algorithms.

### Data analysis

For the determination of the PET parameters the same region of interests (ROI) were used as in our previous publication^5^. In addition to MTV, SUV, and TLG also ASP and SUR were determined for these ROIs. ASP was computed as.

1 $$\mathrm{ASP\, }=\,\sqrt[3]{H}-1\mathrm{ \,with\, H}= \frac{1}{36\pi }\cdot \frac{{S}^{3}}{{MTV}^{2}},$$

where *S* is the surface of the ROI^21,22^. S was computed as the sum of all voxel surfaces that form the outer and inner surfaces of the MTV multiplied by the factor 2/3. Note that this corresponds to the approximation of the surface area of discrete 3D objects using six voxel classes as described by Ref.^30^. Also note that this definition of the MTV surface area is distinctly different from the definition by the Image Biomarker Standardization Initiative (IBSI), and compliance of both definitions cannot be assumed. The IBSI estimates the MTV surface area using a mesh-based representation after triangulation of the MTV’s outer surface^31^.

The arterial blood SUV (BSUV) needed for the computation of SUR values was determined using a convolutional neural network as described by Nikulin et al.^32^. Lesion SUR_max_ was computed as the uptake time corrected ratio of lesion SUV_max_ and BSUV. Uptake time correction to T_0_ = 75 min p.i. was performed as described by van den Hoff et al.^14^. A value of zero for the apparent volume of distribution was assumed (i.e. V_r_ = 0 was used in the correction formula) for the reasons discussed in Ref.^15^. The uptake time corrected SUR is then given by.

2 $${\mathrm{SUR}}_{\mathrm{max}} =\frac{{T}_{0}}{T}\times \frac{lesionSUVmax}{BSUV},$$

where *T* is the actual time of measurement in the respective scan. In the following, we omit the index “max” since only the maximum values are considered. ROI definition and analysis was performed using the ROVER software, version 3.0.62 (ABX, Radeberg, Germany).

### Statistical analysis

The same clinical endpoints as in Ref.^5^ were investigated, namely event free survival (EFS) measured when any disease recurrence (distant or loco-regional) or death occur, overall survival (OS), locoregional control (LRC), and freedom from distant metastases (FFDM) measured from the beginning of the therapy to death and/or event. Median follow up of the survivors was 43.5 months (interquartile range: 22.3–68.4). In 5 case the follow up was below 6 month. The investigated clinical parameters were age, histology, grading, hyperthermia, teleradiotherapy, brachytherapy, hysterectomy, and FIGO stage. The association of the endpoints with ASP and SUR was analyzed using univariate Cox proportional hazard regression in which the PET data were included as metric parameters. PET parameters showing at least a trend for significance in this analysis were further analyzed in the univariate Cox regression using binarized PET parameters. The cutoff values were calculated by minimizing the p-value in univariate Cox regression as described elsewhere^17^. Stability of optimal cutoff values was tested using the bootstrap method (random resampling with replacement, 10^5^ samples). For each sample, a univariate Cox regression was performed where the same cutoff as in the original data was used to define high and low risk groups. Mean (sample averaged) HR and P-value were computed. The fractions of samples yielding p < 0.05 were determined. The probability of survival was computed and rendered as Kaplan–Meier curves. The independence of parameters was analyzed by multivariate Cox regression, where the clinical parameters, which showed a significant effect in univariate analysis, were included as confounding factors Statistical significance was assumed at a P-value of less than 0.05. Statistical analysis was performed with the R language and environment for statistical computing version 4.1.1^33^.

### Institutional review board statement

Ethical review and approval were waived for this study, due to the retrospective nature of this study.

## Conclusion

The alternative parameter ASP has the potential to improve the prognostic value of [^18^F]FDG PET/CT for event-free survival and locoregional control in radically treated cervical cancer patients. Further investigations are necessary to confirm these promising results.

## Data Availability

The data presented in this study are available in article.

## References

[1] Sung H (2021). Global cancer statistics 2020: GLOBOCAN estimates of incidence and mortality worldwide for 36 cancers in 185 countries. CA Cancer J. Clin..

[2] Cohen PA, Jhingran A, Oaknin A, Denny L (2019). Cervical cancer. Lancet.

[3] Wang D (2021). The role of the metabolic parameters of ^18^F-FDG PET/CT in Patients with locally advanced cervical cancer. Front. Oncol..

[4] Narayanan P, Sahdev A (2017). The role of ^18^F-FDG PET CT in common gynaecological malignancies. Br. J. Radiol..

[5] Cegla P (2021). Prognostic value of pretherapeutic primary tumor MTV from [^18^F]FDG PET in radically treated cervical cancer patients. Metabolites.

[6] Cegla P, Burchardt E, Roszak A, Czepczynski R, Kubiak A, Cholewinski W (2019). Influence of biological parameters assessed in [18F]FDG PET/CT on overall survival in cervical cancer patients. Clin. Nucl. Med..

[7] Leseur J (2016). Pre- and per-treatment 18F-FDG PET/CT parameters to predict recurrence and survival in cervical cancer. Radiother. Oncol..

[8] Kitajima K (2014). Prognostic Value of FDG PET Imaging in Patients with Laryngeal Cancer. PLoS ONE.

[9] Aslan H (2021). Prognostic value of ^18^F-FDG PET/CT parameters and histopathologic variables in head and neck cancer. Braz. J. Otorhinolaryngol..

[10] Hamberg L, Hunter G, Alpert N, Choi N, Babich J, Fischman A (1994). The dose uptake ratio as an index of glucose metabolism: Useful parameter or oversimplification?. J. Nucl. Med..

[11] Keyes J (1995). SUV: Standard uptake or silly useless value?. J. Nucl. Med..

[12] Huang S (2000). Anatomy of SUV. Nucl. Med. Biol..

[13] van den Hoff J (2013). The PET-derived tumor-to-blood standard uptake ratio (SUR) is superior to tumor SUV as a surrogate parameter of the metabolic rate of FDG. EJNMMI Res..

[14] van den Hoff J (2014). Correction of scan time dependence of standard uptake values in oncological PET. EJNMMI Res..

[15] Hofheinz F (2016). Comparative evaluation of SUV, tumor-to-blood standard uptake ratio (SUR), and dual time point measurements for assessment of the metabolic uptake rate in FDG PET. EJNMMI Res..

[16] Hofheinz F, Apostolova I, Oehme L, Kotzerke J, van den Hoff J (2017). Test-retest variability in lesion SUV and lesion SUR in 18F-FDG PET: An analysis of data from two prospective multicenter trials. J. Nucl. Med..

[17] Bütof R (2015). Prognostic value of pretherapeutic tumor-to-blood standardized uptake ratio in patients with esophageal carcinoma. J. Nucl. Med..

[18] Hofheinz F (2016). An investigation of the relation between tumor-to-liver ratio (TLR) and tumor-to-blood standard uptake ratio (SUR) in oncological FDG PET. EJNMMI Res..

[19] Bütof R (2019). Prognostic value of SUR in patients with trimodality treatment of locally advanced esophageal carcinoma. J. Nucl. Med..

[20] Hofheinz F (2019). Confirmation of the prognostic value of pretherapeutic tumor SUR and MTV in patients with esophageal squamous cell carcinoma. Eur. J. Nucl. Med. Mol. Imaging.

[21] Apostolova I (2014). Asphericity of pretherapeutic tumour FDG uptake provides independent prognostic value in head-and-neck cancer. Eur. Radiol..

[22] Hofheinz F (2015). Increased evidence for the prognostic value of primary tumor asphericity in pretherapeutic FDG PET for risk stratification in patients with head and neck cancer. Eur. J. Nucl. Med. Mol. Imaging.

[23] Apostolova I (2014). Quantitative assessment of the asphericity of pretherapeutic FDG uptake as an independent predictor of outcome in NSCLC. BMC Cancer.

[24] Apostolova I (2016). The asphericity of the metabolic tumour volume in NSCLC: Correlation with histopathology and molecular markers. Eur. J. Nucl. Med. Mol. Imaging.

[25] Folkert MR (2017). Predictive modeling of outcomes following definitive chemoradiotherapy for oropharyngeal cancer based on FDG-PET image characteristics. Phys. Med. Biol..

[26] Rogasch JM (2020). Validation of independent prognostic value of asphericity of 18F-fluorodeoxyglucose uptake in non–small-cell lung cancer patients undergoing treatment with curative intent. Clin. Lung Cancer.

[27] Jung JH (2017). CONSORT-Independent prognostic value of asphericity of pretherapeutic F-18 FDG uptake by primary tumors in patients with breast cancer. Medicine.

[28] Cheng NM (2018). Heterogeneity and irregularity of pretreatment ^18^F-fluorodeoxyglucose positron emission tomography improved prognostic stratification of p16-negative high-risk squamous cell carcinoma of the oropharynx. Oral Oncol..

[29] Whi W (2020). Relationship of EGFR mutation to glucose metabolic activity and asphericity of metabolic tumor volume in lung adenocarcinoma. Nucl. Med. Mol. Imaging.

[30] Mullikin JC, Verbeek PW (1993). Surface area estimation of digitized planes. Bioimaging.

[31] Zwanenburg A (2020). The image biomarker standardization initiative: standardized quantitative radiomics for high-throughput image-based phenotyping. Radiology.

[32] Nikulin P (2021). A convolutional neural network for fully automated blood SUV determination to facilitate SUR computation in oncological FDG-PET. Eur. J. Nucl. Med. Mol. Imaging.

[33] R Core Team. R: A Language and Environment for Statistical Computing. R Foundation for Statistical Computing, Vienna, Austria. 2021

